# Acute Tension Hydrothorax in a Cirrhotic Patient With Hepatic Hydrothorax Without Ascites

**DOI:** 10.7759/cureus.13941

**Published:** 2021-03-17

**Authors:** Boniface Malangu, Amjad Shaikh

**Affiliations:** 1 Internal Medicine, Rutgers New Jersey Medical School, Newark, USA

**Keywords:** hepatic hydrothorax, tension hydrothorax, cirrhosis, ascites

## Abstract

We describe a case of a 50-year-old man with alcohol cirrhosis status post transjugular intrahepatic portosystemic shunt (TIPS) who presented with dyspnea, refractory hepatic hydrothorax (HH), and no ascites who subsequently developed acute tension hydrothorax (TH). Urgent ultrasound-guided thoracentesis was performed with a significant improvement of symptoms. Further management consisted of a chest tube placement, subsequently removed with a plan for intermittent thoracentesis as needed, diuretic therapy, and salt restriction. HH occurs in 5%-10% of patients with cirrhosis, and TH in these patients is a rare entity that requires prompt recognition and drainage as it may be life-threatening.

## Introduction

Hepatic hydrothorax (HH) refers to the accumulation of transudate, usually >500 mL, in the pleural space in cirrhotic patients with no cardiac, pleural, or pulmonary disease, and it is estimated to occur in 5%-10% of cases [1,2]. It is generally localized to the right side, in approximately 85% of cases, and rarely occurs in the absence of ascites [3,4]. This accumulation of excess fluid in the pleural space can lead to tension hydrothorax (TH), defined as a massive pleural effusion causing a contralateral shift of the mediastinum associated with hemodynamic abnormalities [5-7]. However, TH is an uncommon entity, and it is rarely found in cirrhotic patients without ascites [8,9]. Here we present a case of a cirrhotic patient with refractory HH and no clinically apparent ascites who presented with dyspnea and a large right-sided pleural effusion and subsequently developed TH prompting treatment with bilevel positive airway pressure ventilation and an urgent thoracentesis with the improvement of symptoms.

## Case presentation

A 50-year-old male with a past medical history notable for alcohol cirrhosis decompensated with ascites, hepatocellular carcinoma status post locoregional therapy, and transjugular intrahepatic portosystemic shunt (TIPS) presented with worsening shortness of breath (SOB) for the past two months. He endorsed mild orthopnea and leg swelling. He denied weight loss, fever, chills, cough, hemoptysis, or paroxysmal nocturnal dyspnea. He had no history of heart failure or chronic kidney disease. One week prior to presentation, he underwent thoracentesis twice at an outside hospital after presenting with a similar complaint of dyspnea. He reported that at least one liter was removed on the first tap, though unclear how much was removed on the second. He denied hemoptysis, history of tuberculosis, recent travel, or sick contacts. His home medications included furosemide and spironolactone.

Physical examination revealed a severely obese, lethargic man but in no acute distress. His vitals were temperature of 36.6 °C, blood pressure (BP) of 129/71, heart rate (HR) of 95 beats/min, respiratory rate (RR) of 18 breaths/min, and oxygen saturation of 95% on six liters nasal cannula. Assessment of jugular venous distention was difficult due to the patient’s body habitus. He had normal cardiac sounds with no added sounds. Examination of the lungs revealed diminished breath sounds on the right hemithorax. The abdomen was soft, with no clinical ascites. He had 3+ bilateral lower extremity pitting edema, no palmar erythema. He was alert and oriented to self, time, and place, and neurologic examination was unremarkable.

**Figure 1:**
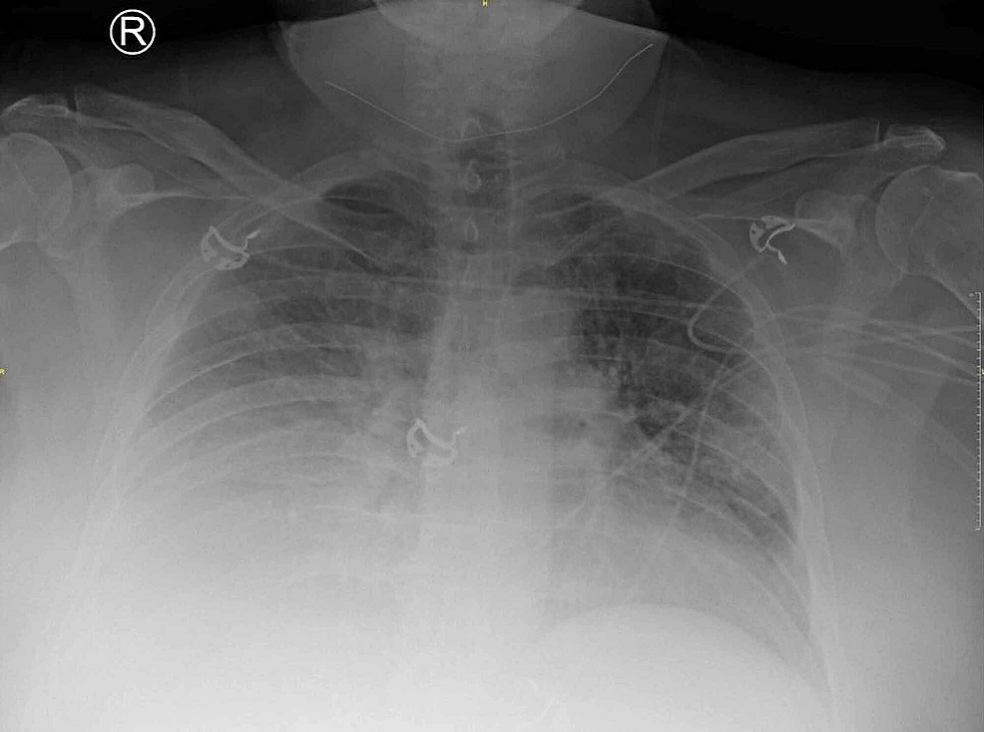
Chest X-ray on presentation Anterior-posterior (AP) view showing opacification of the right hemithorax consistent with right pleural effusion and right basilar atelectasis.

Initial laboratory results revealed hemoglobin of 12.3 g/dL; platelets of 100 x 10x3/ μL; international normalized ratio 1.4; blood urea nitrogen (BUN) 55 mg/dL; creatinine of 1.4 mg/dL (baseline 1 mg/dL); sodium 130 mEq/L; aspartate aminotransferase (AST) 122; alanine aminotransferase (ALT) 128; ammonia 123 umol/L; albumin 2.3 gm/dL. Chest X-ray was remarkable for opacification of the right hemithorax (Figure 1). The patient was admitted with plan to treat aggressively with intravenous diuresis and perform thoracentesis if he did not improve with medical management. 

**Figure 2:**
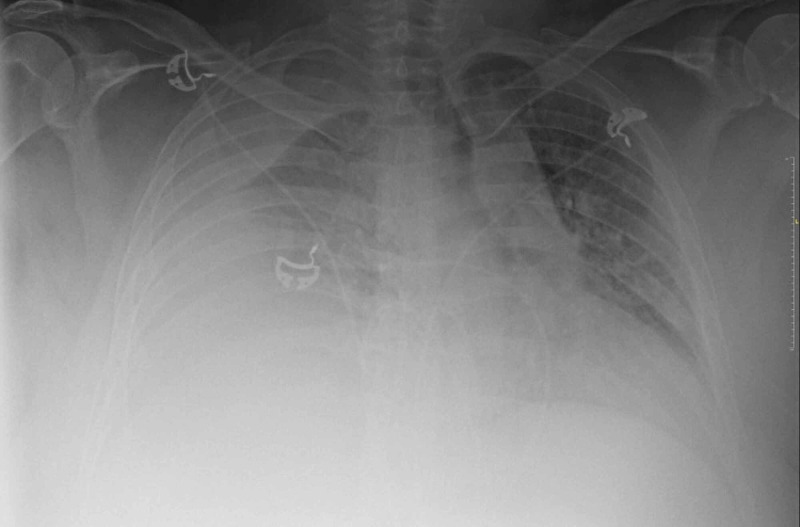
Chest X-ray Anterior-posterior (AP) view showing increased large right pleural effusion with aeration loss of much of the right lung as well as accompanying leftward mediastinal shift.

**Figure 3:**
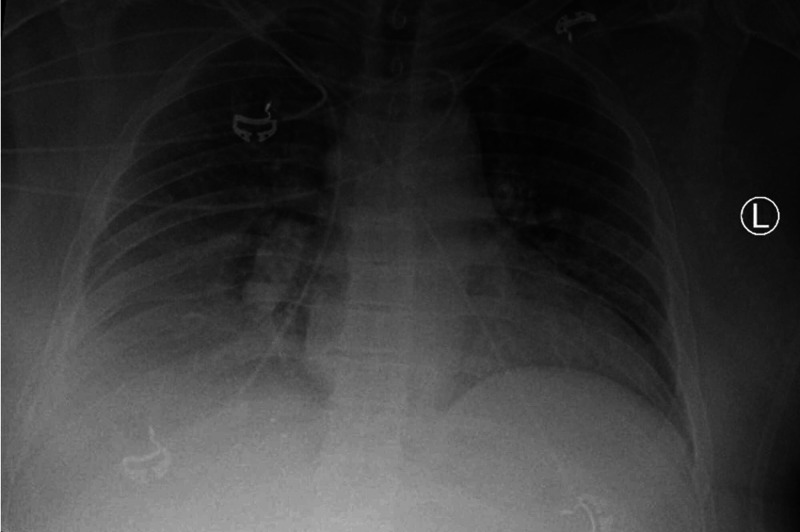
Repeat chest X-ray Anterior-posterior (AP) view, previously seen right pleural effusion has significantly decreased in size. Previously seen mediastinal shift has improved.

On day two of admission, the patient remained hemodynamically stable, and oxygen was titrated down to four liters, which he tolerated well. However, later during the day, the patient began having worsening dyspnea. His vitals were, BP 158/81 mmHg, HR 114 beats/min, and RR of 30 breaths/min. He was increased to bilevel positive airway pressure ventilation, but his symptoms persisted, and arterial blood gas showed a partial pressure of oxygen (PaO2) of 64 on 100% fraction of inspired oxygen (FiO2). Chest radiograph revealed an increased large right pleural effusion with near-complete right lung atelectasis and accompanying leftward mediastinal shift (Figure 2). An urgent ultrasound-guided thoracentesis was performed at the bedside with 1.8 liters of pale-yellow fluid removed. The procedure was halted when the patient started coughing. Postprocedural point of care ultrasound showed improvement of effusion, though significant fluid remained. A repeat chest X-ray showed improvement of the right pleural effusion and mediastinal shift, and the patient reported significant improvement of dyspnea (Figure 3). A repeat thoracentesis was performed several hours later, and 1.5 liters of pale-yellow fluid was removed. Follow up chest X-ray showed partial re-expansion of the right lower lobe with decreased right pleural fluid.

Transudative pleural effusion was confirmed by laboratory analysis of collected pleural fluid, and cytology was negative for malignancy. A subsequent sonographic Doppler study of the liver showed patent TIPS, no ascites. However, slightly slowed flow velocity was noted mid shunt, suggesting a component of TIPS dysfunction. TIPS revision was performed with reduction of portosystemic gradient from 16 mmHg to 9 mmHg. Wide patency of the TIPS shunt and prompt flow into the right atrium was also noted. As the patient continued to require frequent thoracentesis while receiving maximal medical therapy, a 14 french chest tube was inserted on the right side on hospital day 11. It was removed after 11 days as fluid output decreased. He was then continued on diuretic therapy, fluid restriction, and a low salt diet with a plan for intermittent thoracentesis as needed. He was discharged on hospital day 28 with close follow up.

## Discussion

HH usually occurs in cirrhotic patients with ascites, but a few cases have been reported in patients without ascites [10]. The exact mechanisms involved in the accumulation of ascitic fluid in the pleural space is not clearly understood, though it appears to be due to the passage of ascites to the thorax through small diaphragmatic defects via a pressure gradient directed flow [3]. Similarly, in patients with pleural effusion without ascites, before effusion develops, there must be a diaphragmatic defect permitting the passage of ascitic fluid immediately after there is production of ascites, and HH ensues if the rate of production of ascites in the abdominal cavity exceeds the re‐absorption rate in the pleural cavity [3,8,11]. Over time, the massive accumulation of pleural fluid can result in TH presenting with compression of adjacent lung tissues and contralateral shift of mediastinum structures with associated hemodynamic instability due to the significant elevation of intrathoracic pressure. Similar to tension pneumothorax, TH can result in death unless promptly treated. 

Our patient presented with dyspnea and a large pleural effusion. He did not have any clinical ascites and liver ultrasound was also negative for ascites. His pleural effusion further increased resulting in severe dyspnea, tachycardia, tachypnea, and hypoxemia while on six liters nasal cannula. A portable chest X-ray showed a massive pleural effusion on the right side with tracheal deviation to the left. A diagnosis of TH was made, and an urgent ultrasound-guided thoracentesis was performed with significant relief of symptoms.

As previously mentioned, it is likely that the patient has small diaphragmatic defects through which ascitic fluid accumulates in the pleural cavity at a rapid rate since he required multiple therapeutic thoracentesis in less than one month despite optimal medical therapy. A component of TIPS dysfunction likely played a partial role in the recurrence of HH, but it is unlikely to be the main cause as the sonographic Doppler study of the liver did confirm patency, and the patient also continued to accumulate a significant amount of ascitic fluid in the pleural space after TIPS revision requiring insertion of a chest tube for several days.

Sodium restriction and diuretic therapy is the mainstay therapy of HH, and in the case of refractory HH, defined as persistent hydrothorax despite a sodium-restricted diet and diuretic therapy, therapeutic thoracentesis and TIPS are two management options often considered for symptom relief [12]. However, even in the absence of ascites, clinicians should be aware that patients with refractory HH may need close monitoring of fluid status and respiratory status as a rapid accumulation of ascitic fluid in the pleural space may lead to an acute TH, which can lead to a catastrophic outcome if not promptly recognized and treated.

## Conclusions

In summary, we hereby presented a case of a cirrhotic patient who presented with refractory HH in the absence of ascites and subsequently developed TH. While TH rarely occurs in cirrhotic patients without ascites, it is important for clinicians to be cognizant of this complication, especially in patients with refractory HH. This is particularly important as the prompt diagnosis of this uncommon entity and rapid drainage greatly contribute to a favorable outcome. On the other hand, further research is needed to fully elucidate the mechanism involved in the accumulation of ascitic fluid in the pleural space, especially in cirrhotic patients without ascites.
